# pVAX-WIF-1真核表达载体的构建及其抗肺癌作用

**DOI:** 10.3779/j.issn.1009-3419.2015.07.04

**Published:** 2015-07-20

**Authors:** 宁 安, 心梅 罗, 苏娟 叶, 宇 王, 蔚菡 杨, 倩倩 蒋, 文 朱

**Affiliations:** 1 610072 成都，四川省医学科学院，四川省人民医院肿瘤科 Department of Medical Oncology, Sichuan Academy of Medical Sciences and Sichuan Provincial People′ Hospital, 610072 Chengdu, China; 2 610041 成都，四川大学华西医院/生物治疗国家重点实验室 State Key Laboratory of Biotherapy and Cancer Center, West China Hospital, Sichuan University, 610041 Chengdu, China

**Keywords:** 肺癌, *WIF-1*基因, A549, 基因治疗, 真核表达载体, Lung cancer, WIF-1, A549, Gene therapy, Eukaryotic expression vector

## Abstract

**背景与目的:**

*WIF-1*是肺癌中重要的抑癌基因之一，其编码蛋白WIF-1能够通过抑制Wnt/β-catenin信号通路达到抑制肿瘤生长增殖及促进凋亡等抗肺癌作用。本研究旨在利用美国食品和药品管理委员会认可用于临床治疗的pVAX载体，构建pVAX-WIF-1真核表达载体，初步探究pVAX-WIF-1在体内外对A549肺癌细胞的抗肿瘤作用。

**方法:**

应用PCR方法扩增人WIF-1编码序列片段，构建pVAX-WIF-1载体后转染肺癌A549细胞，Western blot检测*WIF-1*基因的表达；采用MTT法、Hoechst3235染色和流式细胞术分别检测pVAX-WIF-1在体外对肺癌A549细胞增殖、凋亡作用的影响；构建A549小鼠皮下瘤模型，探究pVAX-WIF-1在体内的抗肺癌作用。

**结果:**

真核表达质粒载体pVAX-WIF-1构建成功，pVAX-WIF-1转染A549细胞后，WIF-1蛋白表达升高。pVAX-WIF-1转染A549后能抑制细胞增殖和诱导凋亡。动物体内结果表明，pVAX-WIF-1能有效抑制肺癌肿瘤生长。

**结论:**

本研究成功构建了pVAX-WIF-1真核表达质粒，pVAX-WIF-1能明显抑制A549肺癌细胞增殖和促进凋亡，有效抑制A549皮下瘤生长。本研究将为*WIF-1*基因的肺癌临床治疗奠定了基础。

## Abstract (en)

**Background and objective:**

*WIF-1* is an important tumor-suppressing gene in lung cancer, and its encoding protein WIF-1 can reduce proliferation and promote apoptosis by inhibiting Wnt/β-catenin signaling in lung cancer. This study constructs a eukaryotic expression plasmid carrying WIF-1 using FDA-approved clinical plasmid pVAX and explores the anti-tumor effect of pVAX-WIF-1 on A549 lung cancer cells *in vitro* and *vivo*.

**Methods:**

The DNA fragment of human WIF-1 coding sequence was amplified by PCR and was cloned into the multiple cloning sites of eukaryotic expression vector pVAX to construct pVAX-WIF-1. A recombinant plasmid was transfected into lung cancer A549 cells, and the expression of *WIF-1* genes was verified by Western blot after transfection. Subsequently, the effect of pVAX-WIF-1 on cell apoptosis and proliferation was identified by MTT assay, staining A549 cells with Hoechst 3235, and flow cytometry. Finally, the A549 subcutaneous xenograft was established to detect the effect of pVAX-WIF-1 on lung tumor growth *in vivo*.

**Results:**

The results of restriction enzyme digestion, PCR, and sequencing indicated that eukaryotic expression plasmid pVAX-WIF-1 was successfully constructed. The protein expression level of WIF-1 was increased in the transfected A549 cells. Further results showed that transfection with pVAX-WIF-1 significantly inhibited proliferation and promoted apoptosis in A549 cells. Moreover, pVAX-WIF-1 significantly inhibited the tumor growth of the A549 subcutaneous xenograft *in vivo*.

**Conclusion:**

The recombinant eukaryotic expression vector pVAX-WIF-1 was successfully constructed. Transfection with pVAX-WIF-1 could significantly inhibit proliferation and promote apoptosis of lung cancer A549 cells and also effectively inhibit the tumor growth of the A549 subcutaneous xenograft *in vivo*. Our research can contribute to clinical applications of WIF-1 in lung cancer gene therapy.

肺癌是目前全世界发病率和病死率最高的恶性肿瘤。常规的放化疗和手术都难以取得较好的疗效，以致于其5年生存率只有15%-20%。目前认为肺癌发生发展的主要原因之一是由于基因表达异常导致细胞内信号通路紊乱。因此，通过靶向信号通路中的成员来调控异常的信号通路是突破肺癌治疗瓶颈的一种有效的治疗方法。其中，信号通路的特异性拮抗剂已被认为是靶向信号通路异常的极具前景的方法。

Wnt/β-连环蛋白(β-catenin)信号通路，是控制个体生长发育的重要通路之一，参与胚胎发育的全部过程，包括胚胎发生时早期体轴形成、细胞分化及各种器官发育^[1]^。Wnt配体与Frizzled受体结合后引发糖原合成激酶-3β(GSK-3β)从APC/Axin/GSK-3 β复合体移位及后续的一系列反应。当没有接受Wnt信号时，细胞间粘附衔接蛋白或共转录因子(β-catenin)被APC/Axin/GSK-3β复合物靶向性降解。当存在Wnt信号时，Wnt配体与细胞膜上的Fz受体家族及低密度脂蛋白相关蛋白家族的LRP5/6相结合，激活胞内的散乱蛋白(Dishevelled, Dvl)，从而引发GSK-3β从APC/Axin/GSK-3β复合体移位，进一步抑制APC/Axin/GSK-3β复合物降解细胞质中游离的β-catenin，导致β-catenin稳定存在且逐渐累积，进入细胞核后与LEF/TCF等转录因子结合，最终激活Wnt基因^[2]^。目前研究表明，Wnt/β-catenin信号传导通路异常与肺癌的发生、预后、治疗抵抗相关，其中拮抗因子失活是造成肺癌中Wnt/β-catenin异常激活的重要原因^[3]^。

WIF-1是Wnt/β-catenin信号通路重要的拮抗剂之一，其编码蛋白WIF-1通过在细胞外隙与WNT配体结合，抑制Wnt/β-catenin信号通路^[4]^。在肺癌中，Wissmann等^[5]^发现*WIF-1*基因在非小细胞肺癌中因甲基化发生下调的机率为75.8%，其中腺癌占36.4%(4/11)，鳞癌占95.5%(21/22)。此外，Mazieres等^[6]^也发现在18对新鲜肺癌组织中，WIF-1基因启动子甲基化占到83%(15/18)。Korobko等^[7]^也分析了48例肺癌患者，其中67%的肺癌病例中出现WIF-1基因表达下调。因此，WIF-1的失活可能是肺癌中Wnt/β-catenin通路异常激活的重要原因。在已有的关于WIF-1抗肺癌的研究中，Kim等^[8]^利用pcDNA3.1载体构建了pcDNA3.1-WIF-1质粒，通过转染A549和H460细胞，已经证实WIF-1具有促进肺癌细胞的凋亡作用，并且伴随Wnt/β-catenint通路下游靶基因cylcin D1和c-myc的转录下调。而美国食品和药品管理委员会(Food and Drug Admistraton, FDA)批准用于动物实验的载体必须具备卡那抗性，以此来替代pcDNA3.1中的氨苄霉素抗性筛选基因，最大程度地减少抗性筛选基因和人类基因组发生重组的可能性，减少治疗风险^[9]^。因此，为了将来将WIF-1应用于临床的需要，我们将WIF-1的DNA片段连接到含有卡那抗性的pVAX载体上，构建pVAX-WIF-1真核表达质粒，进一步探究pVAX-WIF-1对肺癌细胞的作用影响。

本研究拟构建pVAX-WIF-1真核表达质粒载体，初步探讨pVAX-WIF-1转染A549细胞后WIF-1蛋白表达水平的变化，在体外对肺癌细胞凋亡及增殖的影响，及在体内对肺癌肿瘤生长的抑制作用。本研究旨在为*WIF-1*基因治疗进入临床提供实验基础。

## 1 材料和方法

### 1.1 主要试剂

人质粒pBluescriptR-WIF-1购于美国Open Biosystems公司。真核表达质粒载体pVAX购于美国Invitrogen公司。人肺癌细胞株A549购自美国ATCC(American Type Culture Collection)。大肠杆菌JM109购于美国Clontech公司，由本实验保种。T4 DNA连接酶、HindⅢ和XbaⅠ限制性内切酶购于美国Fermentas公司；PCR Kit、胶回收试剂盒购于日本Takara公司；质粒提取试剂盒购于德国Qiagen公司；Lipofectamine^TM^2000转染试剂购于美国Invitrogen公司；Hoechst 3235购于美国Sigma公司；Anti-WIF-1购于CST公司；HRP标记二抗购于北京中杉金桥生物技术有限公司；化学发光检测试剂盒购自天根生物技术有限公司；BCA蛋白定量试剂盒购于美国Thermo公司；PVDF膜(0.22 μm)购自Millipore公司；其他试剂均为国产分析纯试剂。细胞培养6孔板购于美国Coster公司。雌性免疫缺陷行BALB/c裸鼠(5周龄)购于北京华阜康，饲养于无病原菌的SPF级动物房。阳离子脂质体(Catonic Liposomes, LPs)由本实验化学组制备^[10]^。

### 1.2 pVAX-WIF-1真核表达质粒的构建

取购买的pBluescriptR-WIF-1菌株划平板，次日挑取单克隆，进一步进行扩大培养，然后将培养得到的菌液进行质粒小抽提取。下一步以提取的pBluescriptR-WIF-1质粒为模板，通过PCR的方法，扩增出*WIF-1*基因的编码区序列，经双酶切连接到pVAX载体上，构建含卡那霉素抗性的真核表达质粒pVAX-WIF-1。引物以WIF-1在GenBank中的序列BC018037.1为模板，利用Primer Premier 5.0软件设计：上游引物：5'-GAGGCA AGCTT TGAGCAGCAT GGCCCGGAGGAGCG-3'(酶切位点*Hin*dⅢ)；下游引物：5'-GAAGCTCTAGAC GTTTCAGATGTCGGAGTTCACCAGATGTAATTG-3'(酶切位点*Xba*Ⅰ)。PCR产物长度为1, 188 bp。高保真PCR反应体系按试剂盒说明书进行。PCR扩增条件为：94 ℃变性2 min后进入25个PCR循环(94 ℃、30 s，62 ℃、30 s，72 ℃、3.5 min)，72 ℃、10 min，4 ℃保存。全长*WIF-1*基因序列扩增后，经1%琼脂糖电泳鉴定PCR产物，载体和PCR产物经限制性内切酶*Hin*dⅢ、*Xba*Ⅰ酶切纯化后，定向插入pVAX的多克隆位点，转化JM109感受态细胞，利用卡那霉素筛选阳性克隆，经酶切鉴定正确的克隆由上海Invitrogen公司进行测序。

### 1.3 pVAX-WIF-1真核表达质粒的细胞转染

本实验分为3组：空白组、pVAX组和pVAX-WIF-1组。取对数生长期A549细胞接种于6孔板中，当细胞融合率达到60%时，将pVAX和pVAX-WIF-1质粒分别与Lipofectamine 2000在室温下共孵育20 min后进行瞬时转染，未转染的A549细胞及转染空载体pVAX的细胞作为阴性对照。此步骤按照Lipofectamine 2000说明书进行操作。转染48 h后收集细胞进行后续的试验。

### 1.4 Western blot检测WIF-1蛋白表达水平

以Lipofectamine 2000介导pVAX，pVAX-WIF-1质粒转染A549细胞，方法同上，以未转染的A549细胞和转染pVAX的细胞为阴性对照。48 h后收细胞提取蛋白样品，以BCA法测定蛋白浓度。将等量蛋白通过15%聚丙烯酰胺凝胶电泳分离，电转PVDF膜，电压为86 V，时间90 min。5%脱脂奶粉4 ℃封闭2 h，TBST洗膜，加入一抗(兔抗人WIF-1单抗和鼠抗人β-actin多抗，稀释比例为1:1, 000)，一抗4 ℃孵育过夜，TBST洗膜3次；加入HRP标记的二抗(兔抗鼠IgG 1:5, 000)，37 ℃孵育1 h，TBST洗膜3次，等量的ECL反应液A和B混合后加至膜上，显影。

### 1.5 pVAX-WIF-1真核表达质粒转染对A549细胞增殖的影响

以Lipofectamine 2000介导pVAX、pVAX-WIF-1质粒转染A549细胞，方法同上，以未转染的A549细胞核转染空载体pVAX的细胞为阴性对照。转染后培养48 h，每孔加入20 μL(2 μg/μL)MTT液，孵育4 h，小心吸弃上清，再每孔加入150 μL二甲亚砜(DMSO)，室温下，于微孔板振荡器上振荡10 min-20 min，通过酶标仪检测各孔在波长为492 nm时的吸光度(OD值)，计算出细胞增殖抑制率(inhibitory ratio, IR)。每个组做3个复孔，实验重复3次。

### 1.6 pVAX-WIF-1真核表达质粒转染对A549细胞凋亡的影响

以Lipofectamine 2000介导pVAX、pVAX-WIF-1质粒转染A549细胞，方法同上，以未转染的A549细胞核转染空载体pVAX的细胞为阴性对照。转染后继续培养48 h，每孔加入1 mL新鲜配制的卡诺氏固定液，静置10 min-15 min，弃去固定液，再加入1 mL卡诺氏固定液，静置10 min-15 min。弃去固定液，空气中干燥5 min，加入1 mL 0.5 μg/mL的Hoechest33258避光染色10 min-20 min后，PBS清洗2次-3次，每次5 min，荧光显微镜下观察凋亡细胞形态。

细胞转染后培养48 h，采用AnnexinV-FITC/PI双染色法，通过流式细胞术定量检测转染pVAX-WIF-1对肺癌细胞株凋亡的影响。PBS清洗细胞2次，加入2 mL不含EDTA的胰蛋白酶消化2 min-3 min，加入含2%BSA的PBS终止消化，轻轻将细胞吹打下来。收集细胞悬液至BD管中，计数，1, 300 rpm离心5 min。加入适量含2%BSA的PBS重悬细胞，调整细胞个数至10^6^个，1, 300 rpm离心5 min，弃上清，重复洗涤一次。加入300 μL Binding buffer重悬细胞，再加入3 μL AnnexinV-FITC混匀，避光染色5 min，后加入3 μL PropidiumIodide混匀制成单细胞悬液，室温避光染色5 min-15 min，用流式细胞仪检测细胞凋亡。

### 1.7 pVAX-WIF-1真核表达质粒转染在体内对肺癌生长作用影响

以5×10^6^ cell/mouse的A549肺癌细胞100 µL无血清培养基悬液对BALB/c裸鼠进行皮下注射，建立皮下瘤模型。当肿瘤体积长至(50-100)mm^3^(平均直径约5 mm-8 mm)左右时，将裸鼠随机分成3组：Glucose组、pVAX组和pVAX-WIF-1组，每组4只开始治疗。质粒DNA和脂质体分别用5%的葡萄糖水稀释，按1:4(w/w)的比例复合。每只小鼠给药20 μg质粒，给药体系100 µL，进行瘤内注射治疗。治疗期间每周测量瘤体两次，瘤体体积按照V (mm^3^)= 0.52 × length(mm)× width^2^(mm^2^)公式计算，直到治疗结束两周后，处死小鼠，剥离皮下瘤组织，拍照记录。

### 1.8 统计学方法

各组实验独立重复3次，实验数据用SPSS 13.0统计软件进行分析，实验组与对照组的组间差异采用方差分析，以*P* < 0.05为差异有统计学意义。

## 2 结果

### 2.1 pVAX-WIF-1真核表达质粒的构建、鉴定和测序

为构建pVAX-WIF-1真核表达质粒载体，首先以提取的质粒pBluescriptR-WIF-1为模板，利用Primer Premier 5.0软件设计克隆引物，克隆WIF-1全长基因片段，PCR产物经琼脂糖凝胶电泳结果表明，WIF-1片段大小正确(大小为1, 188 bp)。将该WIF-1 PCR产物片段和pVAX载体直接进行*Hin*dⅢ和*Xba*Ⅰ双酶切。WIF-1经*Hin*dⅢ和*Xba*Ⅰ酶切后约为1, 176 bp。pVAX载体经*Hin*dⅢ和*Xba*Ⅰ双酶切后由环状结构(大小为3, 000 bp)变为2, 920 bp的单链结构。然后将两酶切产物纯化后经T4连接酶进行连接，最后得到真核表达质粒载体pVAX-WIF-1(大小为4, 086 bp)。经初步电泳筛选正确后，将构建的pVAX-WIF-1质粒载体进行PCR和双酶切鉴定。PCR产物经琼脂糖凝胶电泳结果显示，WIF-1片段大小约为1, 188 bp；双酶切产物经琼脂糖凝胶电泳结果显示，pVAX-WIF-1质粒载体经*Hin*dⅢ和*Xba*Ⅰ双酶切后得到1, 176 bp和2, 920 bp左右两条带，与预期结果一致(图 1)。阳性克隆测序和比对结果显示真核表达质粒载体pVAX-WIF-1构建成功，该载体含有WIF-1全长编码序列且无碱基突变。

**1:**
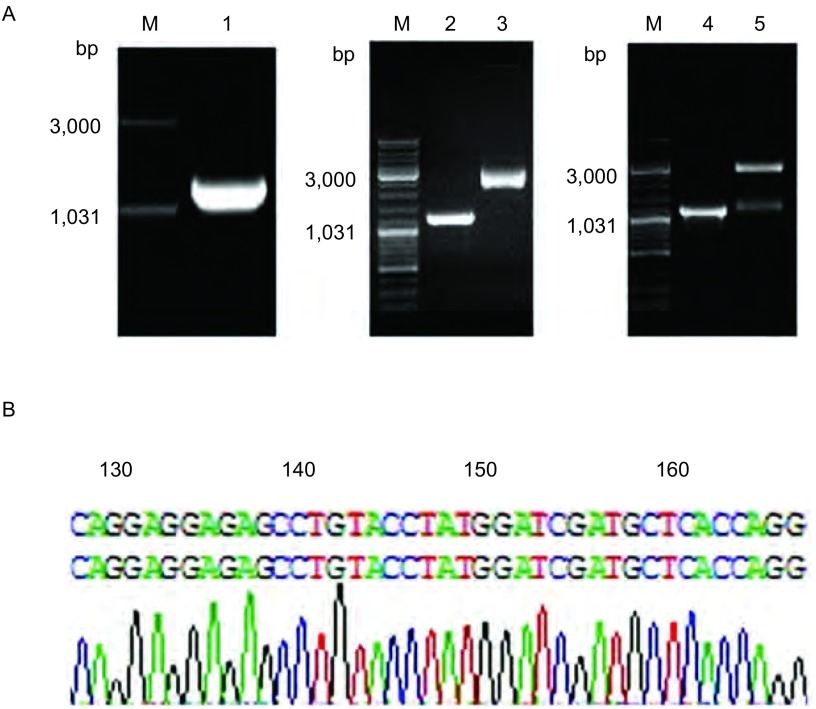
真核表达质粒pVAX-WIF-1的构建。A：质粒pVAX-WIF-1的构建。M：Marker(DL2000)；1：WIF-1 PCR产物；2：WIF-1 PCR产物经*Hin*dⅢ和*Xba*Ⅰ切割并纯化；3：载体pVAX经*Hin*dⅢ和*Xba*Ⅰ切割并纯化；4：阳性克隆的PCR鉴定；5：阳性克隆的双酶切鉴定；B：阳性克隆的测序鉴定(因序列过长，此处只显示以起始码开始的部分测序结果)。 Construction of eukaryotic expression plasmid pVAX-WIF-1. A: Construction of pVAX-WIF-1 plasmid. M: Marker(DL2000); 1: PCR products of WIF-1; 2: The digested and purified PCR products; 3: The digested and purified pVAX; 4: The positive clones confirmed by PCR; 5: The positive clones confirmed by double restriction enzyme digestion; B: Sequencing analysis of the positive clones (Part of the sequence).

### 2.2 pVAX-WIF-1转染后A549细胞中WIF-1的表达检测

以Lipofectamine 2000介导pVAX，pVAX-WIF-1质粒分别转染A549细胞，以未转染的A549细胞及转染空载pVAX的A549细胞为阴性对照。48 h后分别提取各处理组A549细胞的总蛋白，Western blot检测结果显示转染pVAX-WIF-1后，WIF-1蛋白在A549细胞中的表达上调，其相对分子质量约为42 kDa，与预期大小相符(图 2)。

**2:**
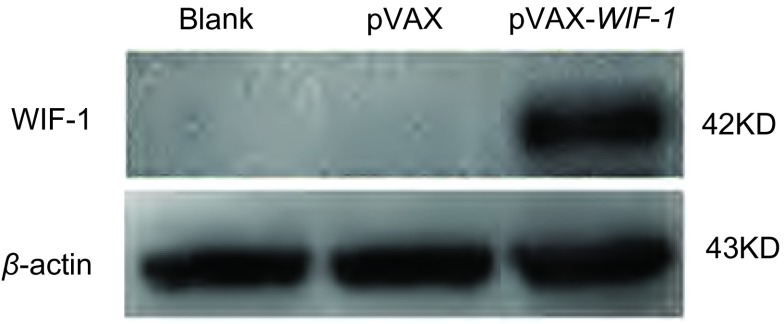
Western blot检测A549细胞转染pVAX-WIF-1 48 h后*WIF-1*基因的蛋白表达水平 Protein expression level of WIF-1 in A549 cells transfected with pVAX-WIF-1 for 48 h by Western blot analysis

### 2.3 pVAX-WIF-1转染对肺癌细胞A549增殖的影响

为了研究pVAX-WIF-1对A549细胞增殖的影响，我们将Lipo2000包裹pVAX-WIF-1基因后转染A549细胞。本实验分为空白、pVAX、pVAX-WIF-1组，pVAX-WIF-1转染A549细胞48 h后，加入MTT检测。结果显示，在A549细胞株中，pVAX-WIF-1对细胞增殖的抑制率为55%，与空白和空载比较，pVAX-WIF-1转染后可明显抑制肺癌细胞A549的增殖(图 3，*P* < 0.01)。

**3:**
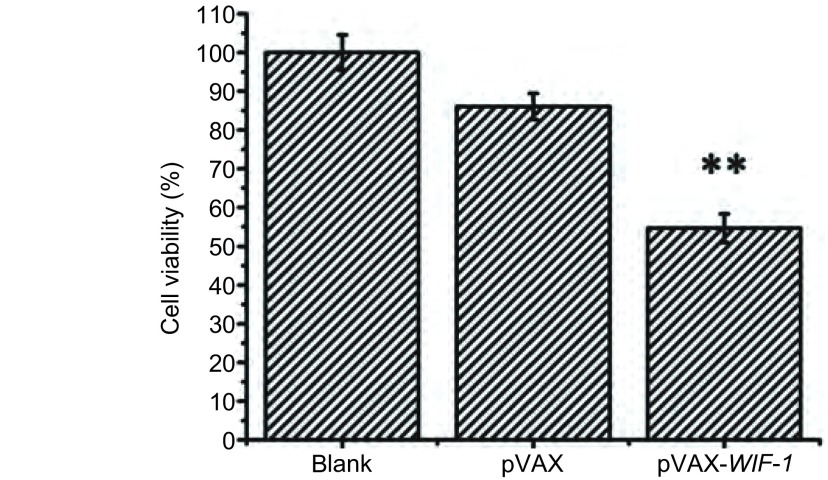
MTT法检测转染pVAX-*WIF-1*基因对A549细胞生长的影响。^**^：*P* < 0.01。 Inhibition of cell proliferation of A549 cells after transfection with pVAX-WIF-1 by MTT assay. ^**^: *P* < 0.01.

### 2.4 pVAX-WIF-1转染对肺癌细胞A549凋亡检测

为探究pVAX-WIF-1对A549细胞凋亡作用的影响，我们将Lipofectamine 2000包裹pVAX-WIF-1后转染A549细胞，细胞转染48 h后经Hoechst3235染色，通过电子荧光显微镜观察可见，pVAX-WIF-1转染的细胞在紫外光激发时发出明亮的蓝色荧光，核出现固缩裂解为碎块，产生明显的凋亡小体(图 4A)；而转染了空载体或未转染的细胞生长状态良好，少见凋亡小体。进一步，将Lipofectamine 2000包裹pVAX-WIF-1后转染A549细胞，48 h后使用流式细胞仪检测，结果显示：空白组和空载组的细胞凋亡率分别为10%和21%，而转染pVAX-WIF-1组的细胞凋亡率达到了62%(图 4B和图 4C，*P* < 0.01)。以上结果表明pVAX-WIF-1质粒在体外具有明显诱导肺癌细胞A549凋亡的作用。

**4:**
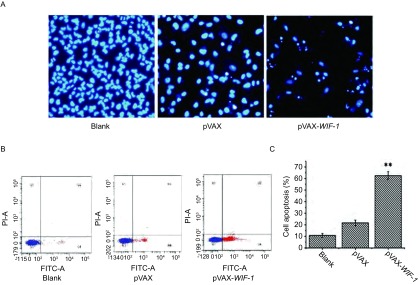
*WIF-1*基因转染A549细胞48 h后对细胞凋亡的影响。A：Hoechest3235染色检测A549细胞转染pVAX-WIF-1的细胞凋亡形态；B：流式细胞术检测*WIF-1*基因对A549细胞凋亡的影响；C：流式数据统计细胞凋亡率。^**^: *P* < 0.01。 Induction of apoptosis of A549 cells transfected with pVAX-WIF-1 for 48 h. A: Detection of A549 cell apoptosis by Hoechst staining; B: Detection of A549 cell apoptosis by FCM; C: Cell apoptosis ratio of lung cancer A549 cells by FCM. ^**^: *P* < 0.01.

### 2.5 pVAX-WIF-1转染抑制皮下瘤生长检测

为探究pVAX-WIF-1质粒在体内的抗肺癌的作用，我们建立了A549裸鼠皮下瘤模型，将脂质体包裹pVAX-WIF-1后，进行瘤内注射治疗。如图 5A所示，在pVAX-WIF-1治疗组中，WIF-1在瘤体内的表达升高。其治疗结果表明，Glucose组和pVAX组在给药治疗过程中，小鼠皮下瘤生长较快，而经pVAX-WIF-1治疗后的小鼠皮下瘤生长速度明显降低(图 5B)。治疗结束后，取小鼠皮下瘤测量其瘤体体积，pVAX-WIF-1组与Glucose组相比，其皮下瘤生长抑制率达50%(图 5C，*P* < 0.01)。以上结果说明，pVAX-WIF-1在体内对肺癌的生长也有明显的抑制作用。而在治疗期间，对各组小鼠的体重变化观察结果显示，各组间和组内的小鼠体重差异较小，提示pVAX-WIF-1脂质体复合物对荷瘤小鼠没有明显毒性(图 5D，图 5E)。

**5:**
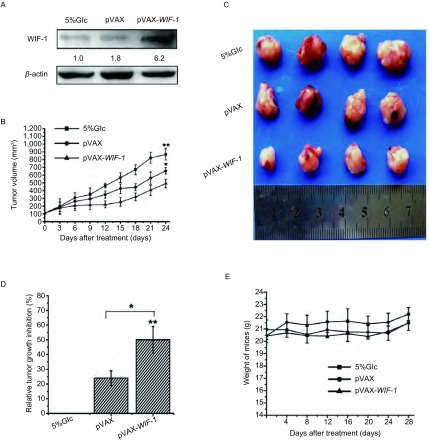
pVAX-WIF-1对小鼠皮下瘤的抑制效果。A：pVAX-WIF-1在皮下瘤瘤内的表达情况；B：皮下瘤瘤体治疗期间生长曲线；C：治疗结束后剥离的小鼠皮下瘤；D：皮下瘤生长相对抑制率；E：皮下瘤小鼠治疗期间体重变化。^*^：*P* < 0.05，^**^：*P* < 0.01。 Tumor growth inhibition of pVAX-WIF-1 *in vivo*. A: The expression of pVAX-WIF-1 in subcutaneous tumor; B: The curve of subcutaneous tumor volume; C: The picture of subcutaneous tumors peeled from mice after treatment; D: Relative growth inhibition of subcutaneous tumor; E: Subcutaneous tumor mice body weight curve. ^*^: *P* < 0.05; ^**^: *P* < 0.01.

## 3 讨论

肺癌在全世界范围内是发病率和病死率最高的恶性肿瘤，其中抑癌基因的失活导致细胞信号通路过度激活，使细胞增殖失控，恶性转化，是肺癌发生发展的主要原因。因此，通过靶向抑癌基因，调控异常信号通路是一种极具前景的肿瘤治疗方法。

近年来，基于Wnt/β-catenin信号通路在肿瘤中的普遍异常激活的现象，已有研究^[11]^表明，通过Wnt/β-catenin信号通路的特异性拮抗剂对其进行调控可能是抗肿瘤治疗的一个新的方法。目前有研究^[12-17]^报道，在鼻咽癌细胞、大肠癌细胞、黑色素瘤细胞、前列腺瘤细胞、肝癌细胞、骨肉瘤细胞中转染WIF-1后，均能通过下调Wnt/β-catenin信号通路，抑制肿瘤细胞的生长。在肾癌细胞中发现，转染WIF-1后，诱发肿瘤细胞凋亡并伴随Wnt/β-catenin信号通路被下调^[18]^。在宫颈癌细胞中转染WIF-1也观察到了诱导细胞凋亡的现象^[19]^。此外，通过转染WIF-1均能在体内抑制前列腺瘤和骨肉瘤小鼠皮下瘤的生长^[15, 17]^。同样，在肺癌中，Kim等^[8]^利用pcDNA3.1载体构建了pcDNA3.1-WIF-1质粒，通过转染A549和H460细胞，发现WIF-1具有下调Wnt/β-catenin信号通路抑制肺癌细胞生长和促进细胞凋亡的作用。这些研究均表明，转染WIF-1能够通过下调Wnt/β-catenin信号通路抑制包括肺癌等多种肿瘤生长和促进凋亡，因而为WIF-1的临床应用提供了理论依据。

在本研究中，我们通过高保真PCR扩增WIF-1目的片段，并成功构建了真核表达质粒载体pVAX-WIF-1。通过阳离子脂质体转染的方法将pVAX-WIF-1和pVAX转染A549肺癌细胞，在蛋白水平检测到该基因表达上调；经MTT法检测WIF-1转染对肺癌A549细胞株生长活性的影响，发现*WIF-1*基因转染后可明显抑制A549细胞的生长增殖。经Hoechst染色和Annexin-V联合PI双染经流式细胞术检测*WIF-1*基因转染诱导了A549细胞凋亡。在进一步的体内实验中，pVAX-WIF-1表现出有效抑制皮下瘤生长的作用。这些结果均说明构建的pVAX-WIF-1可以有效抑制肺癌细胞的生长增殖，促进肺癌细胞凋亡。本研究中运用的基因载体质粒是FDA推荐的唯一可以应用于人体实验的载体质粒，且质粒本身具有真核基因表达调控序列，强启动子pCMV启动子和poly A信号，可以在哺乳动物细胞中高水平表达重组蛋白等特点^[9]^。因此，本研究为将来*WIF-1*基因应用于肺癌的临床治疗奠定了实验基础。

## References

[1] Nusse R (2012). Wnt signaling. Cold Spring Harb Perspect Biol.

[2] Clevers H, Nusse R (2012). Wnt/beta-catenin signaling and disease. Cell.

[3] Stewart DJ (2014). Wnt signaling pathway in non-small cell lung cancer. J Natl Cancer Inst.

[4] Malinauskas T, Aricescu AR, Lu W (2011). Modular mechanism of Wnt signaling inhibition by Wnt inhibitory factor 1. Nat Struct Mol Biol.

[5] Wissmann C, Wild PJ, Kaiser S (2003). WIF1, a component of the Wnt pathway, is down-regulated in prostate, breast, lung, and bladder cancer. J Pathol.

[6] Mazieres J, He B, You L (2004). Wnt inhibitory factor-1 is silenced by promoter hypermethylation in human lung cancer. Cancer Res.

[7] Korobko E, Kalinichenko S, Shepelev M (2007). Suppression of the WIF1 transcript and protein in non-small cell lung carcinomas. Mol Genet Microbiol.

[8] Kim J, You L, Xu Z (2007). Wnt inhibitory factor inhibits lung cancer cell growth. J Thorac Cardiov Sur.

[9] Qu YP, Liu JG, Yang DQ (2009). The application of eukaryotic expression vector pVAX. Wenzhou Yi Xue Yuan Xue Bao.

[10] Ou W, Ye S, Yang W (2012). Enhanced antitumor effect of cisplatin in human NSCLC cells by tumor suppressor LKB1. Cancer Gene Ther.

[b11] 11Takebe N, Lum L, Ivy SP. Wnt signaling in cancer pathogenesis and therapeutics. In: Frank DA, ed. Signaling pathways in cancer pathogenesis and therapy. New York: Springer, 2012: 81-94.

[12] Lin YC, You L, Xu Z (2006). Wnt signaling activation and WIF-1 silencing in nasopharyngeal cancer cell lines. Biochem Bioph Res Commun.

[13] Taniguchi H, Yamamoto H, Hirata T (2005). Frequent epigenetic inactivation of Wnt inhibitory factor-1 in human gastrointestinal cancers. Oncogene.

[14] Lin YC, You L, Xu Z (2007). Wnt inhibitory factor-1 gene transfer inhibits melanoma cell growth. Hum Gene Ther.

[15] Yee D, Tang Y, Li X (2010). The Wnt inhibitory factor 1 restoration in prostate cancer cells was associated with reduced tumor growth, decreased capacity of cell migration and invasion and a reversal of epithelial to mesenchymal transition. Mol Cancer.

[16] Deng Y, Yu B, Cheng Q (2010). Epigenetic silencing of WIF-1 in hepatocellular carcinomas. J Cancer Res Clin Oncol.

[17] Rubin EM, Guo Y, Tu K (2010). Wnt inhibitory factor 1 decreases tumorigenesis and metastasis in osteosarcoma. Mol Cancer Ther.

[18] Kawakami K, Hirata J, Yamamura S (2009). Functional significance of Wnt inhibitory factor-1 gene in kidney cancer. Cancer Res.

[19] Ramachandran I, Thavathiru E, Ramalingam S (2012). Wnt inhibitory factor 1 induces apoptosis and inhibits cervical cancer growth, invasion and angiogenesis *in vivo*. Oncogene.

